# Local feature acquisition and global context understanding network for very high-resolution land cover classification

**DOI:** 10.1038/s41598-024-63363-7

**Published:** 2024-06-01

**Authors:** Zhengpeng Li, Jun Hu, Kunyang Wu, Jiawei Miao, Zixue Zhao, Jiansheng Wu

**Affiliations:** 1 School of Electronic and Information Engineering, University of Science and Technology Liaoning, Anshan, China; 2 Liaoning Province Key Laboratory of Intelligent Construction and Internet of Things Application Technologies, Anshan, China; 3 College of Instrumentation and Electrical Engineering, Jilin University, Changchun, China; 4 National Geophysical Exploration Equipment Engineering Research Center, Jilin University, Changchun, China; 5 Key Laboratory of Geophysical Exploration Equipment Ministry of Education of China (Jilin University), Changchun, China; 6 School of Computer Science and Software Engineering, University of Science and Technology Liaoning, Anshan, China

**Keywords:** Very high-resolution remote sensing imagery, Land cover, Vision transformer, Local feature acquisition, Global context understanding, Computer science, Scientific data, Environmental sciences

## Abstract

Very high-resolution remote sensing images hold promising applications in ground observation tasks, paving the way for highly competitive solutions using image processing techniques for land cover classification. To address the challenges faced by convolutional neural network (CNNs) in exploring contextual information in remote sensing image land cover classification and the limitations of vision transformer (ViT) series in effectively capturing local details and spatial information, we propose a local feature acquisition and global context understanding network (LFAGCU). Specifically, we design a multidimensional and multichannel convolutional module to construct a local feature extractor aimed at capturing local information and spatial relationships within images. Simultaneously, we introduce a global feature learning module that utilizes multiple sets of multi-head attention mechanisms for modeling global semantic information, abstracting the overall feature representation of remote sensing images. Validation, comparative analyses, and ablation experiments conducted on three different scales of publicly available datasets demonstrate the effectiveness and generalization capability of the LFAGCU method. Results show its effectiveness in locating category attribute information related to remote sensing areas and its exceptional generalization capability. Code is available at https://github.com/lzp-lkd/LFAGCU.

## Introduction

In recent years, the advancement of very high-resolution (VHR) satellite sensors has profoundly impacted the domain of multi-image processing. VHR satellite sensors enable the acquisition of earth's surface imagery at exceptionally high resolutions, offering substantial contributions to domains such as earth resource understanding and management^1,2^, urban planning^3^, and environmental monitoring^4^. Notably, the utilization of VHR imagery has marked a significant breakthrough in the context of land cover classification tasks^5,6^. While the CNNs have showcased impressive capabilities in accurately categorizing land cover through intricate feature extraction from satellite and aerial images, their inherent receptive fields are inherently confined. This constraint can impede the effective capture of extensive geographical features embedded in remote-sensing images. Moreover, traditional convolutional methodologies encounter challenges when handling high-resolution images, primarily due to their struggle in accurately identifying subtle spatial patterns and spectral variabilities. This assertion stems from the inherent complexity present in high-resolution imagery, encompassing intricate details and intricate spatial structures that conventional convolutional approaches may overlook or inadequately capture, thus leading to diminished performance in processing such images.

The quantity of VHR imagery is significantly smaller than that of natural environment images. Classic CNNs often enhance accuracy by increasing the number of convolutional layers, using smaller convolution kernels, and incorporating multiple residuals^7,8^. However, this approach to increasing network depth and complexity can exacerbate overfitting issues, especially in situations with limited data where models might overfit to training data excessively. To address the issues brought about by deepening the network with additional convolutional layers, researchers have adopted attention mechanisms for global modeling of VHR images to compensate for the CNNs' limitation in capturing only local features during hierarchical learning. In the field of remote sensing, the ViT has become a focal point of research due to its immense potential in global information modeling. This breakthrough is largely attributed to the ViT series models' learning methods based on attention mechanisms. Wang et al.^9^ effectively augment the shortcomings of CNNs in learning local features by using attention mechanisms to globally model very high-resolution imagery. However, the ViT structure tends to rely on global self-attention mechanisms which fail to adequately extract and utilize local features in remote sensing imagery, leading to decreased performance in tasks that require handling of local detail information, such as geospatial classification in remote sensing images.

An increasing number of researchers are exploring the potential applications of a combined mechanism of CNNs and Transformers in VHR land cover classification imagery^10,11^. Ding et al.^12^ proposed utilizing multiscale feature fusion and probabilistic decision fusion strategies to integrate local spatial features with global spectral features, addressing complex spatial-spectral associations and facilitating effective interaction between multimodal data. Song et al.^13^ designed a dual-backbone attention fusion module and a multilayer dense connectivity network to integrate both local and global contextual information. To overcome the limitations inherent in convolution operations which restrict the network’s ability to extract global contextual information, and the Transformer’s deficiencies in capturing detailed local information, this paper proposes a local feature acquisition and global context understanding network. This approach attempts to fully couple the hierarchical feature representation of CNNs with the global dependency relationships of Transformers, leveraging the strengths of both paradigms.

The primary contributions of this research are as follows.

1. Proposing a neural network, LFAGCU, for VHR remote sensing image classification, which synergistically learns the potential of local–global semantic information embedded in images through two design paradigms.
2. Constructing a local feature extractor that explicitly considers spatial relationships between fine-grained image pixels and the characteristics of geographic attributes using CNN biases and inductive techniques.
3. Introducing a global feature learning module (GFL) for image modeling, facilitating a proficient understanding of spatial relationships and inherent contextual clues in terrestrial entities.
4. Conducting a series of experiments on widely-used open-source datasets, namely RSCCN7, WHU-RS19, and UCMerced-LandUse, demonstrating that LFAGCU outperforms other advanced methods for remote sensing image classification.

## Related works

### Feature extractor based on CNN

Rezaee et al.^14^ integrated spatial features and spectral attributes using a pre-trained AlexNet, enhancing thematic land cover information. Jamali et al.^15^ highlighted the synergy between wavelet transformation and deep convolutional networks for effective feature extraction from imagery. Scott et al.^16^ merged CaffeNet, GoogLeNet, and ResNet50 architectures, focusing on category-specific information aggregation. Residual networks like ResNet have found extensive use in land cover analysis; however, in limited dataset scenarios, they may face overfitting issues^17^. Jamali et al.^18^ emphasized tailored network architectures for comprehensive land feature attribute capture in land cover tasks. Singh^19^ combined convolutional structures and contractive-expansive-contractive networks with residual connections for improved feature representation. Conventional CNN models might not suit VHR satellite sensor-acquired land cover images due to acquisition disparities. Scholars aim for multi-scale and multi-modal learning for remote sensing insights. Gbodjo et al.^20^ used knowledge distillation to handle multi-temporal and multi-scale remote sensing data, enriching feature representations. Li et al.^21^ fused optical and imagery, enhancing interpretability with a semantic consistency constraint algorithm. Ye et al.^22^ proposed controllable filters and a multi-scale strategy for improved remote sensing feature learning. Fan et al.^23^ applied "pyramid features of orientated self-similarity" for multi-modal remote sensing image matching, overcoming geometric distortions and intensity variations.

The current research focuses on the pivotal role of CNNs in the field of land cover classification. However, these methods do not adequately consider the representation of global features from a macro perspective, especially when it comes to tasks involving the capture and presentation of semantic information in VHR remote sensing imagery.

### Feature extractor based on transformer

The recent progress in deep models, especially the ViT^24^, has sparked interest in leveraging attention mechanisms for land cover classification. Li et al.^25^ utilized a multi-head encoder and a knowledge-guided decoder to capture diverse land patterns. However, ViT faces challenges due to its high parameter count compared to traditional CNNs^26,27^. Lv et al.^28^ introduced a spatial channel-preserved ViT model, enhancing feature preservation and representing a significant advancement. Yao et al.^29^ demonstrated exploiting spatial and pattern-specific channel information by integrating ViT modules with separable convolutional architectures. Zhao et al.^30^ explored the multi-sample contrastive ViT, revealing nuanced patterns across distinct samples. Hou et al.^31^ enriched cross-domain learning using pseudo-label self-training and consistency regularization, highlighting ViT's adaptability in addressing diverse challenges. Tang et al.^32^ employed a self-attention mechanism to integrate features at different levels, deeply exploring subtle representations in remote sensing images.

Recent ViT-based methods, leveraging attention mechanisms, have made significant strides in image processing, particularly in handling high-resolution images. However, these methods often suffer from a high number of parameters, leading to increased model complexity. While ViT methods focus on capturing global semantic features of high-resolution images, their effectiveness in learning subtle local features is not as prominent as CNN models, primarily due to the lack of an inductive bias strategy. Addressing this challenge, we propose an approach that combines local induction and global feature integration to complement the deficiencies of ViT in learning local features.

## Methodology

**Figure 1:**
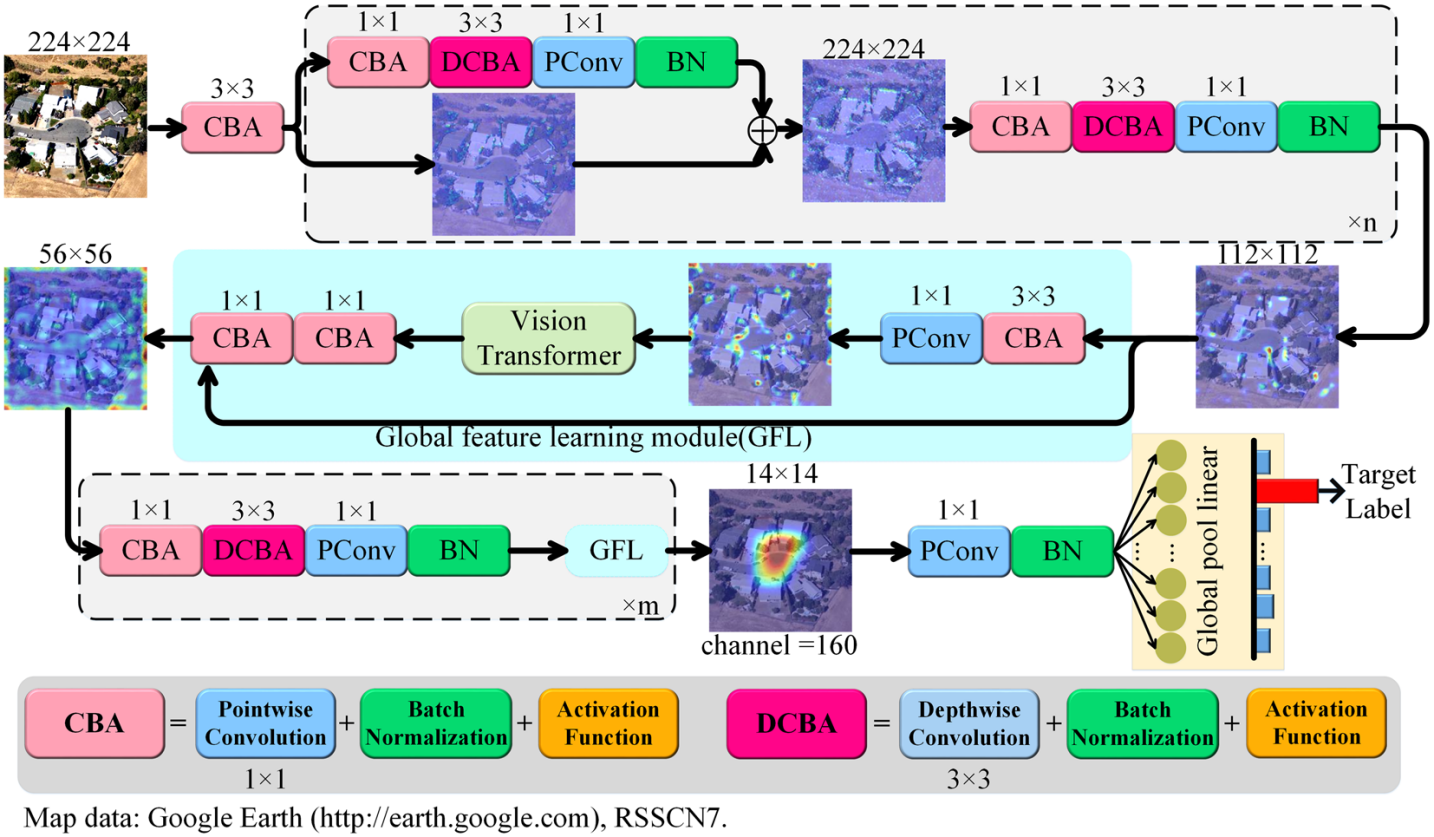
Overview of the local perception and global context modeling network.

We introduce LFAGCU, a new network design specifically crafted for accurately classifying land cover in VHR remote sensing images. This architecture combines classical convolutions, pointwise convolutions, and focused depthwise convolutions (DConv), which inherently capture local sensitivities. Additionally, we incorporate a GFL to understand comprehensive semantic attributes and spatial relationships. As illustrated in Fig. 1, LFAGCU takes inspiration from the ResNet series paradigm, employing a multi-residual connection approach to preserve the innate features extracted within each feature extraction unit. These original features are amalgamated with subsequent updated features to foster an enhanced grasp of both local and global attributes inherent in land objects. The overarching LFAGCU framework comprises $n = 2$ sets of linearly combined convolutional groups, strategically devised to fully capture the contextual relationships and geometric intricacies underpinning the entirety of the land entities' spatial landscape. In tandem, $m = 2$ sets of GFL are harnessed to holistically model the entire image, allowing the features at each position to comprehensively perceive the broader array of inter-land relationships. These modules dynamically adjust the feature weights at each position based on the spatial relationships between land objects, thereby adeptly encapsulating the global characteristics of land entities. Ultimately, the synthesis of contextually enriched and spatially encoded features culminates via a global pooling layer, culminating in the definitive classification of each pixel's category within the input image. This innovative design of LFAGCU aims to seamlessly integrate local features, global attributes, and spatial relationships within the context of land cover classification tasks, thus enhancing classification accuracy and the capacity for feature expression in the realm of VHR remote sensing imagery.

### Local perception

The central focus of LFAGCU lies in local feature extraction from VHR remote sensing images, achieved through the integration of two pivotal modules: convolution-batch normalization-activation (CBA) and DConv-batch normalization-activation (DCBA). In the structural framework, for a given input image, a preliminary preprocessing step converts it into a three-dimensional tensor with dimensions *H* representing height, *W* representing width, and C signifying the number of channels. Within the initial CBA layer, the mathematical representation of the output stemming from the standard convolutional layer can be expressed as follows:

1 $$ Y(i,j,k) = \sum\limits_{a = 0}^{n - 1} {\sum\limits_{b = 0}^{n - 1} {\sum\limits_{c = 0}^{C - 1} {X_{(i + a,j + b,c)} \cdot \omega_{(a,b,c,k)} } } } , $$

where $Y \in {\mathbb{R}}^{H \times W \times K}$ represents the resulting output tensor, while *K* stands for the count of convolutional kernels. The index k is used to denote a specific convolutional kernel. Furthermore, the indices *i* and *j* respectively represent the dimensions of height and width in the resulting convolved tensor. The variables a and b are employed to indicate the offsets in the vertical and horizontal directions of the convolutional kernel on the input tensor. Additionally, the variable c signifies the index of the channel in the input tensor. $\omega_{(a,b,c,k)}$ denotes the weight associated with the convolutional kernel at the position (*a*, *b*), connecting channel *c* in the input tensor to channel *k*.

**Figure 2:**
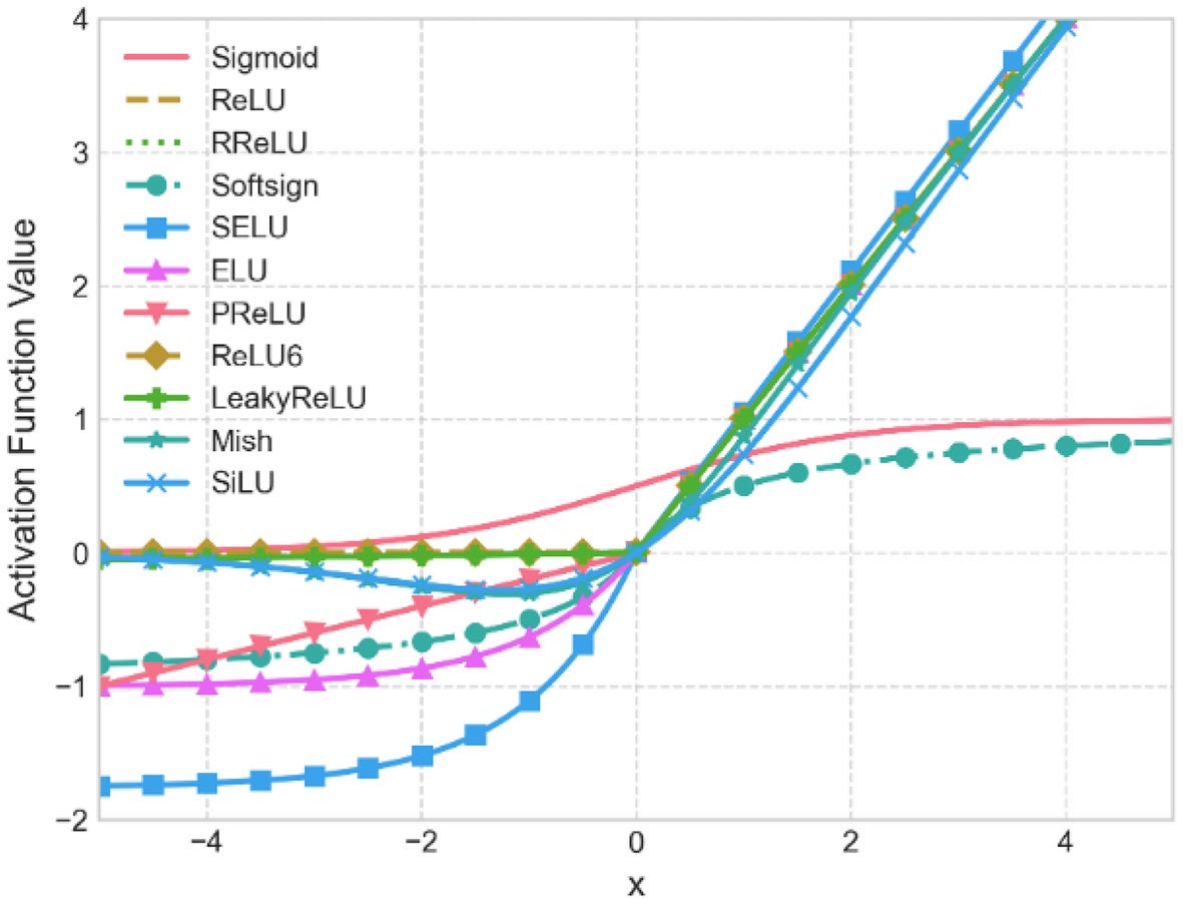
Nonlinear transformation function curves.

In the domain of VHR image processing, it is often imperative for models to possess sufficient capacity to intricately capture the nuances present in the images. However, this pursuit can potentially lead to the problem of overfitting, especially in scenarios where the available data is limited^18^. Moreover, VHR remote sensing images are subject to significant variability due to variations in geographical regions, temporal factors, and sensor characteristics^33^. To address these challenges, LFAGCU incorporates standard *n* × *n* convolutions paired with batch normalization layers (BN). This integration of BN aids in normalizing the mean and variance of individual feature channels, effectively aligning them with a standard normal distribution. This normalization process fosters enhanced learning of pertinent feature representations by rendering the network more amenable to capturing the intricacies embedded within VHR images. The mathematical formulation for the output of BN can be succinctly described as:

2 $$ Y^{BN} = \gamma \cdot \frac{X - \mu }{{\sqrt {\sigma^{2} + \varepsilon } }} + \beta , $$

where $Y^{BN}$ is the normalized output feature map, $\gamma$ and $\beta$ are learnable parameters, $\gamma$ is used for scaled normalized features, $\beta$ is used for translated normalized features, $\mu$ is the mean value of *X*, $\sigma^{2}$ is the variance of *X*, and $\varepsilon$ is a positive parameter. Following the feature map normalization, LFAGCU incorporates a mechanism for introducing nonlinearity. VHR remote sensing images often exhibit intricate terrain boundaries, textures, and distinctive features, necessitating the network's capability to capture diverse nonlinear characteristics inherent in the data^34^. In Fig. 2, we present a visual comparison of the distinct nonlinear transformation curves associated with eleven diverse nonlinear activation functions. Through meticulous experimental comparisons and subsequent discussions (elaborated in Sect. "Comparative experiment and analysis"), we observe that the sigmoid-weighted linear unit (SiLU) activation function excels within the LFAGCU framework. The SiLU activation function amalgamates both linear and nonlinear attributes, endowing it with enhanced adaptability to intricate feature variations evident in VHR remote sensing images. This selection is a product of comprehensive contemplation and exhaustive experimental validation. We firmly contend that the SiLU activation function adeptly facilitates LFAGCU in capturing pivotal image features, thereby elevating the performance of land cover classification tasks. The mathematical formulation of SiLU is as follows:

3 $$ Y^{s} = X \cdot \frac{1}{{1 + e^{ - X} }}. $$

To enhance the learning of variations in the channel dimension, we employ pointwise convolution on the feature map resulting from the standard convolution, as shown Eq. (4). This involves computing convolution operations at each pixel location of the feature map, ensuring that spatial dimensions remain unchanged. By combining information from different channels with the introduced nonlinearity, the tensor is projected into a higher-dimensional space, resulting in a more intricate feature representation.

4 $$ Y_{i,j,c}^{pc} = \sum\limits_{d = 1}^{D} {X \cdot \omega_{1,1,d,c} } , $$

where $Y_{i,j,c}^{pc}$ represents the value of channel *c* at position (*i*, *j*) in the output feature map. $\omega_{1,1,d,c}$ denotes the weight of the pointwise convolution, connecting input channel *d* to output channel *c*.

Finally, LFAGCU incorporates DConv (as shown Eq. (5)), a technique that applies individual convolution kernels to each channel of the input feature map. This approach facilitates the maintenance of channel separability while capturing spatial dependencies among channels. Given an input feature map X, DConv performs separate convolutions using *n* × *n* kernels for each input channel, resulting in distinct sets of output features.

5 $$ Y_{i,j,d}^{DC} = \sum\limits_{p = 1}^{n} {\sum\limits_{q = 1}^{n} {X_{i + (p - 1),j + (q - 1),d} } } \cdot \omega_{p,q,d} , $$

where $Y_{i,j,d}^{DC}$ represents the value at position (*i*, *j*) in the output feature map, in channel *d*. $X_{i + p,j + (q - 1),d}$ stands for the value at position $(i + (p - 1),j + (q - 1))$ in the input feature map, also in channel *d*. $\omega_{p,q,d}$ corresponds to the weight associated with channel *d* at position (*p*,*q*) of the convolutional kernel. For the feature map obtained after convolution, individual convolutional kernels are applied to each input channel. This implementation yields a form of channel-wise separable convolution operation, enabling the decoupling of information between different channels.

### Global context modeling

By incorporating deeper convolutional layers and expanding the dimensions of the sliding window, the capability to effectively extract features from VHR images can be enhanced, thereby bolstering the network's adaptability for tasks such as land cover classification. However, this approach results in a substantial increase in the model's trainable parameters, potentially compromising its practical deployment due to increased computational demands and memory requirements. To address this challenge, LFAGCU introduces the (GFL) that aims to ensure the modeling of long-range non-local dependencies by leveraging an effective receptive field spanning the dimensions *H* × *W*. Notably, while ViT has demonstrated remarkable effectiveness in diverse computer vision tasks^35,36^, it presents limitations in terms of spatial inductive bias and its propensity for fine-tuning, hindering its full potential for certain tasks^37^. To overcome the limitations of weighted averaging of each pixel within the receptive field during convolution operations, which can lead to noise pixels affecting the distinguishability of image target pixels, the GFL module utilizes a multi-head self-attention mechanism for comprehensive global context modeling. This enables a better capture of long-range non-local dependencies, thereby ensuring the preservation of spatial information for each patch within the feature map.

**Figure 3:**
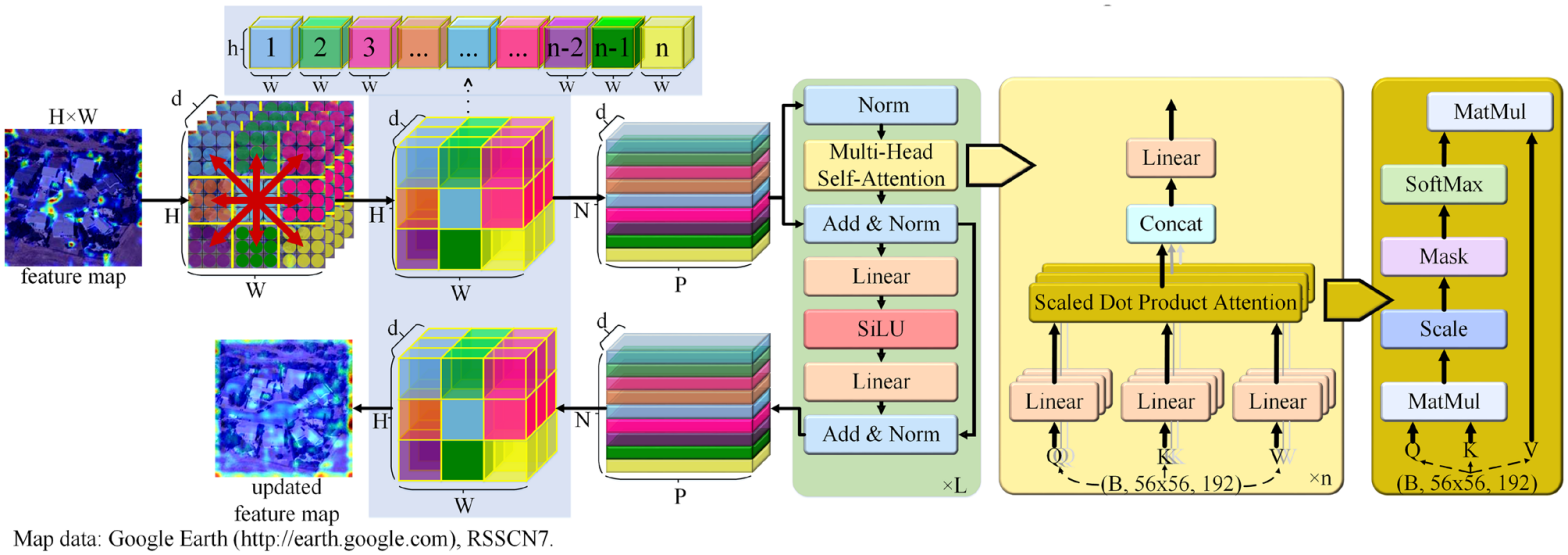
Overview of the global feature learning module.

As illustrated in Fig. 3, the GFL unfolds the feature map post pointwise convolution into non-overlapping flattened patches, denoted as $X_{pc} \in {\mathbb{R}}^{P \times N \times d}$. Here, *P* represents the total number of patches, *N* signifies the patch dimensionality, and d stands for the feature depth. This approach aims to enable effective information aggregation across patches while preserving their spatial relationships. Where *P* = *wh* and *N* = *HW*, *h* and *w* respectively represent the height and width of each patch. Additionally, *N* corresponds to the total number of elements within a patch, which is determined by the product of the patch height (*h*) and width (*w*). This configuration enables efficient organization of information for subsequent analysis and processing. In the framework of the multi-head self-attention mechanism, the flattened patch tensor $Xp \in {\mathbb{R}}^{P \times N \times d}$ is multiplied with the weight matrices $W_{q}$, $W_{k}$, and $W_{v}$. This operation yields linear transformations *Q*, *K*, and* V*, corresponding to query, key, and value representations, respectively. Within each attention head, the dot product between queries and keys is computed, followed by a scaling step to regulate the magnitude of attention scores. The resulting attention scores matrix is denoted as $Attention_{{head_{i} }}$.

6 $$ Attention_{{head_{i} }} = softmax\,\,\left( {\frac{{Q \times K^{T} }}{\sqrt d }} \right). $$

The value matrix $V_{i}$ is multiplied element-wise by the attention score matrix $Attention_{{head_{i} }}$ and subsequently summed, yielding the output matrix $O_{{head_{i} }}$ specific to each attention head.

7 $$ O_{{head_{i} }} = Attention_{{head_{i} }} \cdot V_{i} . $$

In the multi-head self-attention layer of GFL, each attention head generates a self-attention matrix, which encapsulates the significance weights attributed to different positions within the remote sensing image. These weights are instrumental in computing a weighted average of features within the flattened patch tensor, facilitating the assimilation of global contextual insights. Ultimately, by concatenating the individual head output matrices, denoted as $O_{{head_{i} }}$, a consolidated multi-head self-attention output matrix $O \in {\mathbb{R}}^{{P \times N \times \left( {K \times d} \right)}}$ is obtained. This amalgamated matrix empowers the model to encapsulate the interrelationships and interdependencies among diverse image positions. Notably, the multi-head self-attention strategy employed by LFAGCU is underpinned by a localized connectivity approach, whereby each patch position selectively focuses on its proximate counterparts. This strategic orientation contributes to a more refined assimilation of local features and spatial patterns in the image. This strategic localization augments the model's acuity to nuances, thereby enabling a more nuanced comprehension of subtle disparities and intricate configurations in the image.

In summation, GFL adeptly harnesses the potential of the multi-head self-attention mechanism to ascertain both local and global feature representations while upholding the integrity of the patch and pixel sequence. The harmonization of conventional convolutions and transformative elements in the LFAGCU architecture facilitates a localized and comprehensive interpretation of VHR remote sensing images. Concurrently, the model's acumen to discern subtle intricacies and intricate relationships within the image is accentuated.

### Loss function

In the context of land cover classification tasks, remote sensing images often exhibit significant variations across different categories, giving rise to a challenging scenario exacerbated by the presence of imbalanced multi-class training samples. This challenge bears paramount importance, as model training could be disproportionately influenced by the prevalence of samples from certain dominant categories, potentially resulting in compromised classification performance on underrepresented minority classes. In light of this, in order to provide more effective guidance for the optimization of LFAGCU, we posit the model's output as a predictive probability distribution denoted by $P = (p_{1} ,p_{2} ,...,p_{C} )$, where C represents the cardinality of the class set, and $p_{i}$ denotes the probability assigned by the model to the sample's membership in the *i*-th class. The actual ground truth labels are encoded as $Y = (y_{1} ,y_{2} ,...,y_{C} )$, where assumes binary values (0 or 1), signifying the true class membership of the sample. The formulation of the loss function is articulated as follows:

8 $$ Loss = - \sum\limits_{i = 1}^{C} {y_{i} \log (p_{i} )} . $$

By minimizing the cross-entropy loss, the model undergoes iterative parameter adjustments during the training process to align its predicted probability distribution with the true label distribution as closely as possible. This endeavor facilitates enhanced model adaptation to diverse category samples, thereby improving its classification performance across various classes.

## Materials and data preparation

### Network training

In the training process, we employed NVIDIA 3080ti GPU and conducted training for 80 epochs. The batch size was set to 64. During the optimization phase of the network model, we employed the AdamW optimizer with a weight decay (L2 regularization) weight of 1E-2. Furthermore, an initial learning rate of 2E-4 was configured, and a cosine annealing learning rate scheduler was applied to dynamically adjust the model's learning rate. We set a 2-layer local feature extraction block and a 2-layer GFL. We randomly selected 80% of annotated samples from each category to form the training set, allocating 10% for validation. The remaining 10% of the data was reserved as the test set for evaluating model performance. Throughout the model training process, our loss function employed the supervised loss function defined in Eq. (8), guiding the model optimization process. By employing the aforementioned configurations and training strategies, we aimed to fine-tune the LFAGCU model, enhancing its performance and generalization capabilities in the context of land cover classification tasks.

### Study area

In the context of this research, we employed three prominent datasets sourced from the realm of remote sensing imagery, namely the RSSCN7 dataset^38^, the WHU-RS19 dataset^39^, and the UC Merced landuse dataset^40^, to serve as foundational pillars in substantiating the outcomes of our study. A comprehensive tabulation encapsulating data categories, sample sizes, and data dimensions for each of the aforementioned datasets is meticulously documented in Table 1.

**Table 1:** Multi-class land cover datasets.

	Number of sample classes	Image type	Number of images	Image size	Image spatial resolution
RSSCN7	7	Aerial RGB	2800	400 × 400	n/a
WHU-RS19	19	Aerial RGB	1005	600 × 600	0.5 m
UC Merced	21	Aerial RGB	2100	256 × 256	0.3 m

**Figure 4:**
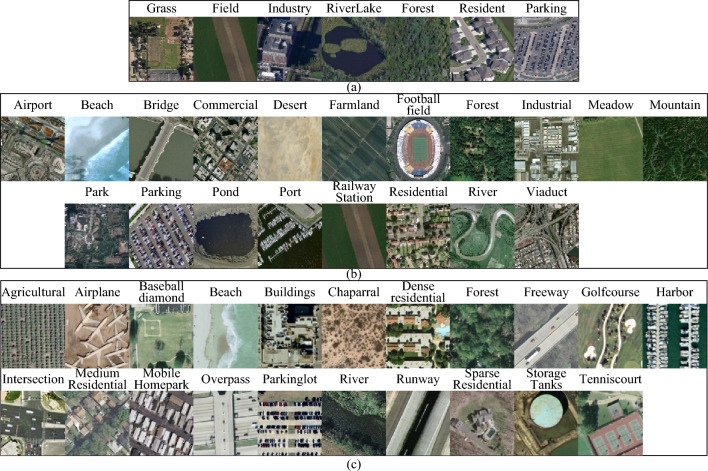
Illustration of experimental data samples. Map data: google earth, RSSCN7 in (**a**), WHU-RS19 in (**b**), and UCMerced-LandUse in (**c**).

The RSSCN7 dataset has emerged as a valuable resource within the realm of remote sensing, incorporating diverse imagery collected from various geographical regions across China. This dataset has found applications in pivotal areas such as scene classification and object recognition^41,42^. It encompasses a wide spectrum of environments including urban, rural, and natural landscapes, thereby encapsulating a rich variety of land features. The dataset encompasses seven prevalent land cover categories as shown in Fig. 4a.

The WHU-RS19 dataset is a prominent resource originating from the Institute of Remote Sensing at Wuhan University, specifically curated for advancing research in the realms of semantic segmentation and land cover classification, as elucidated by Ref.^39,43^. The WHU-RS19 dataset standardizes its evaluation using nineteen prevalent land cover categories, including but not limited to roads, buildings, water bodies, forests, and croplands. Each category is distinguished by its unique combination of spectral and textural attributes. This intricate interplay of spectral information and textural nuances underscores the dataset's intrinsic capacity for discerning and classifying diverse land cover classes. A visual schematic of the research categories encapsulated within the WHU-RS19 dataset is artistically presented in Fig. 4b.

The UC Merced Land Use dataset comprises high-resolution remote sensing images from various regions across the United States, catering to the needs of land use classification tasks^44,45^. The dataset encompasses a diverse range of land use types, including urban, industrial, and agricultural areas. The benchmark dataset encompasses twenty-one distinct land use categories, such as residential zones, airports, highways, and orchards, each characterized by unique color and texture attributes. An illustrative example of the research area within the UCMerced dataset is provided in Fig. 4c.

## Results and discussions

### Comparative experiment and analysis

To ensure a fair comparison, we quantitatively assessed the classification performance of these models using four widely recognized evaluation metrics: average precision (Pre), average recall (Rec), average accuracy (Acc), and average f1 score (F1). We have selected classical models and state-of-the-art land cover classification models for comparative experiments, as shown in Tables 2, 3, and 4. The upper section displays models primarily based on CNN architectures, including neural networks that utilize full CNNs for feature extraction: VGGNet^46^, GoogleNet^47^, ResNet^48^, AlexNet^14,49^, Mobilenet^50^, Shufflenet-v2^51^, densenet^52^, Efficientnet^53^, and the latest models re-evaluating inductive biases and aggregating feature extraction techniques, such as Convnext^54^, Vgg-Vote^55^, DFAGCN^56^, SNN-VGG-15^42^. The middle section includes models based on transformer architectures: MLLD^57^, HSL-MINet^58^, ViT-b-p16^24^, ViT-b-p32^24^, ViT-l-p16^24^, and T2T-VIT-12^59^. The lower section includes models with global–local perspective structures, such as TransResUNet^60^, BPECN^61^, SKAL-AlexNet^62^, SKAL-ResNet18^62^, SKAL-GoogleNet^62^, GCSANet^63^, EMTCAL^32^, and SF-MSFormer-ResNet18^64^.

**Table 2:** The results of different models on the RSSCN7 dataset.

Method	Pre	Rec	Acc	F1
ResNet34^48^^†^	0.8253	0.7588	0.7590	0.7450
ResNet50^48^^†^	0.7864	0.7147	0.7230	0.6861
ResNet101^48^^†^	0.8072	0.7228	0.7266	0.6793
AlexNet^14^^†^	0.7938	0.7866	0.7878	0.7870
VGG11^46^^†^	0.8515	0.8470	0.8489	0.8452
VGG13^46^^†^	0.8393	0.8326	0.8237	0.8252
VGG16^46^^†^	0.8342	0.8216	0.8237	0.8224
VGG19^46^^†^	0.7583	0.7410	0.7374	0.7382
GoogleNet^47^^†^	0.8588	0.8602	0.8597	0.8582
Mobilenetv2^50^^†^	0.9027	0.9012	0.8993	0.9000
Mobilenetv3-l^50^^†^	0.8923	0.8937	0.8921	0.8906
Mobilenetv3-s^50^^†^	0.8421	0.8436	0.8417	0.8399
Shufflenet-0.5^51^^†^	0.9345	0.9374	0.9353	0.9343
Shufflenet-1^51^^†^	0.9632	0.9654	0.9640	0.9639
Shufflenet-1.5^51^^†^	0.9410	0.9439	0.9424	0.9413
Shufflenet-2^51^^†^	0.9674	0.9686	0.9676	0.9675
densenet121^52^^†^	0.9566	0.9570	0.9568	0.9565
densenet161^52^^†^	0.9713	0.9710	0.9712	0.9706
densenet169^52^^†^	0.9636	0.9641	0.9640	0.9635
densenet201^52^^†^	0.9557	0.9584	0.9568	0.9566
Efficient-b0-1k^53^^†^	0.9449	0.9451	0.9460	0.9444
Efficient-b1-1k^53^^†^	0.9396	0.9394	0.9388	0.9388
Efficient-b2-1k^53^^†^	0.9347	0.9350	0.9353	0.9343
Efficient-b3-1k^53^^†^	0.9633	0.9650	0.9640	0.9638
Efficient-b4-1k^53^^†^	0.9643	0.9650	0.9640	0.9643
Efficient-b5-1k^53^^†^	0.9400	0.9412	0.9388	0.9370
Efficient-b6-1k^53^^†^	0.9342	0.9380	0.9353	0.9347
Efficient-b7-1k^53^^†^	0.8786	0.8766	0.8777	0.8748
Efficientv2-l-1k^53^^†^	0.9310	0.9317	0.9317	0.9308
Efficientv2-m-1k^53^^†^	0.9242	0.9249	0.9245	0.9239
Efficientv2-s-1k^53^^†^	0.8908	0.8922	0.8921	0.8902
Convnext-s-1k^54^^†^	0.9403	0.9387	0.9388	0.9382
Convnext-b-22k^54^^†^	0.9438	0.9423	0.9424	0.9415
Convnext-b-1k^54^^†^	0.9527	0.9535	0.9532	0.9529
Convnext-t-1k^54^^†^	0.9665	0.9680	0.9676	0.9667
Convnext-l-1k^54^^†^	0.9450	0.9415	0.9424	0.9414
Convnext-l-22k^54^^†^	0.9608	0.9599	0.9604	0.9599
Convnext-xl-22k^54^^†^	0.9086	0.9065	0.9029	0.9021
DFAGCN^56^^‡^	–	–	0.9414	–
SNN-VGG-15^42^^‡^	–	–	0.9454	–
ViT-b-p16^24^^†^	0.9464	0.9486	0.9460	0.9471
ViT-b-p32^24^^†^	0.9089	0.9095	0.9101	0.9091
ViT-l-p16^24^^†^	0.9571	0.9579	0.9568	0.9574
TransResUNet^60^^†^	0.9654	0.9325	0.9338	0.9256
BPECN^61^^‡^	–	–	0.9400	–
SKAL-AlexNet^62^^‡^	–	–	0.9335	–
SKAL-GoogleNet^62^^‡^	–	–	0.9575	–
SKAL-ResNet18^62^^‡^	–	–	0.9604	–
LFAGCU (ours)	**0.9820**	**0.9820**	**0.9820**	**0.9818**

Bold indicates the optimal solution, ^‡^represents the data results from the reference literature, and ^†^represents the experimental results based on the settings of this paper’s parameters.

**Table 3:** The results of different models on the WHU-RS19 dataset.

Method	Pre	Rec	Acc	F1
AlexNet^14^^†^	0.6031	0.5908	0.5833	0.5591
VGG11^46^^†^	0.8024	0.7904	0.7708	0.7666
VGG13^46^^†^	0.7955	0.8096	0.7812	0.7781
VGG16^46^^†^	0.8704	0.8509	0.8438	0.8446
VGG19^46^^†^	0.8341	0.8412	0.8333	0.8184
GoogleNet^47^^†^	0.8518	0.8228	0.8125	0.8153
ResNet34^48^^†^	0.9089	0.8421	0.8333	0.8371
ResNet50^48^^†^	0.8956	0.8509	0.8646	0.8395
ResNet101^48^^†^	0.8614	0.7728	0.7604	0.7578
Mobilenetv2^50^^†^	0.9895	0.9829	0.9792	0.9848
Mobilenetv3-l^50^^†^	0.9722	0.9671	0.9688	0.9661
Mobilenetv3-s^50^^†^	0.9912	0.9868	0.9896	0.9877
Shufflenet- × 0.5^51^^†^	0.9529	0.9566	0.9479	0.9505
Shufflenet- × 1^51^^†^	0.9868	0.9912	0.9896	0.9877
Shufflenet- × 1.5^51^^†^	0.9836	0.9803	0.9792	0.9800
Shufflenetv2- × 2^51^^†^	0.9912	0.9868	0.9896	0.9877
densenet121^52^^†^	0.9895	0.9868	0.9896	0.9866
densenet161^52^^†^	0.9895	0.9868	0.9896	0.9866
densenet169^52^^†^	0.9912	0.9868	0.9896	0.9877
densenet201^52^^†^	0.9868	0.9868	0.9896	0.9850
Efficient-b0-1k^53^^†^	0.9675	0.9715	0.9688	0.9660
Efficient-b1-1k^53^^†^	0.9781	0.9763	0.9792	0.9743
Efficient-b2-1k^53^^†^	0.9866	0.9789	0.9792	0.9797
Efficient-b3-1k^53^^†^	0.9722	0.9741	0.9688	0.9705
Efficient-b4-1k^53^^†^	0.9942	0.9934	0.9896	0.9934
Efficient-b5-1k^53^^†^	0.9868	0.9868	0.9896	0.9850
Efficient-b6-1k^53^^†^	0.9942	0.9884	0.9879	0.9913
Efficient-b7-1k^53^^†^	0.9538	0.9465	0.9479	0.9465
Efficientv2-l-1k^53^^†^	0.9825	0.9759	0.9688	0.9771
Efficientv2-m-1k^53^^†^	0.9876	0.9846	0.9792	0.9855
Efficientv2-s-1k^53^^†^	0.9412	0.9368	0.9271	0.9284
Convnext-s-1k^54^^†^	0.9749	0.9697	0.9688	0.9694
Convnext-b-22k^54^^†^	0.9688	0.9658	0.9688	0.9634
Convnext-b-1k^54^^†^	0.9925	0.9895	0.9896	0.9901
Convnext-t-1k^54^^†^	0.9722	0.9697	0.9688	0.9677
Convnext-l-1k^54^^†^	0.9810	0.9803	0.9792	0.9784
Convnext-l-22k^54^^†^	0.9732	0.9803	0.9688	0.9743
Convnext-xl-22k^54^^†^	0.9807	0.9737	0.9792	0.9743
MLLD^57^^‡^	–	–	0.9069	–
HSL-MINet^58^^‡^	–	–	0.9122	–
ViT-b-p16^24^^†^	0.9810	0.9803	0.9792	0.9784
ViT-b-p32^24^^†^	0.9722	0.9671	0.9688	0.9661
ViT-l-p16^24^^†^	0.9912	0.9868	0.9896	0.9877
TransResUNet^60^^†^	0.9656	0.9566	0.9583	0.9568
BPECN^61^^‡^	–	–	0.9820	–
SF-MSFormer-ResNet18^64^‡	–	–	0.9860	–
LGFormer	–	–	**0.9920**	–
LFAGCU (ours)	**0.9942**	**0.9934**	0.9896	**0.9934**

Bold indicates the optimal solution, ^‡^represents the data results from the reference literature, and ^†^represents the experimental results based on the settings of this paper's parameters.

**Table 4:** The results of different models on the UCMerced-LandUse dataset.

Method	Pre	Rec	Acc	F1
AlexNet^14^^†^	0.5729	0.5835	0.5681	0.5529
VGG11^46^^†^	0.7477	0.7225	0.7277	0.7188
VGG13^46^^†^	0.8406	0.8365	0.8451	0.8344
VGG16^46^^†^	0.8499	0.8441	0.8498	0.8365
VGG19^46^^†^	0.7596	0.7598	0.7653	0.7448
GoogleNet^47^^†^	0.8622	0.8593	0.8638	0.8521
ResNet34^48^^†^	0.9197	0.8835	0.8826	0.8782
ResNet50^48^^†^	0.9147	0.8772	0.8873	0.8765
ResNet101^48^^†^	0.8976	0.8349	0.8498	0.8283
Mobilenetv2^50^^†^	0.9503	0.9483	0.9484	0.9475
Mobilenetv3-l^50^^†^	0.9198	0.9161	0.9202	0.9147
Mobilenetv3-s^50^^†^	0.9424	0.9320	0.9343	0.9314
Shufflenet- × 0.5^51^^†^	0.9240	0.9241	0.9249	0.9194
Shufflenet- × 1^51^^†^	0.9758	0.9764	0.9765	0.9740
Shufflenet- × 1.5^51^^†^	0.9689	0.9654	0.9671	0.9655
Shufflenetv2- × 2^51^^†^	0.9788	0.9692	0.9718	0.9724
densenet121^52^^†^	0.9897	0.9848	0.9859	0.9865
densenet161^52^^†^	0.9877	0.9828	0.9859	0.9843
densenet169^52^^†^	0.9794	0.9757	0.9765	0.9763
densenet201^52^^†^	0.9804	0.9761	0.9765	0.9777
Efficient-b0-1 k^53^^†^	0.9601	0.9573	0.9577	0.9572
Efficient-b1-1 k^53^^†^	0.9884	0.9884	0.9859	0.9879
Efficient-b2-1 k^53^^†^	0.9893	0.9881	0.9859	0.9879
Efficient-b3-1 k^53^^†^	0.9873	0.9841	0.9859	0.9847
Efficient-b4-1 k^53^^†^	0.9781	0.9765	0.9765	0.9762
Efficient-b5-1 k^53^^†^	0.9728	0.9700	0.9718	0.9697
Efficient-b6-1 k^53^^†^	0.9784	0.9750	0.9765	0.9760
Efficient-b7-1 k^53^^†^	0.9078	0.8725	0.8685	0.8668
efficientV2_l^53^^†^	0.9422	0.9321	0.9343	0.9344
efficientV2_m^53^^†^	0.9201	0.9115	0.9155	0.9099
efficientV2_s^53^^†^	0.9397	0.9272	0.9296	0.9298
Vgg-Vote^55^^‡^	0.9485	0.9642	0.9512	0.9567
Convnext-s-1k^54^^†^	0.9603	0.9615	0.9624	0.9587
Convnext-b-22k^54^^†^	0.9665	0.9701	0.9671	0.9656
Convnext-b-1k^54^^†^	0.9740	0.9728	0.9718	0.9711
Convnext-t-1k^54^^†^	0.9687	0.9669	0.9671	0.9660
Convnext-l-1k^54^^†^	0.9814	0.9816	0.9812	0.9805
Convnext-l-22k^54^^†^	0.9699	0.9630	0.9624	0.9631
Convnext-xl-22k^54^^†^	0.9695	0.9705	0.9671	0.9678
DFAGCN^56^^‡^	–	–	0.9848	–
SNN-VGG-15^42^^‡^	–	–	0.9914	–
MLLD^57^^‡^	–	–	0.7776	–
HSL-MINet^58^^‡^	–	–	0.8189	–
ViT-b-p16^24^^†^	0.9592	0.9568	0.9577	0.9555
ViT-b-p32^24^^†^	0.9697	0.9606	0.9624	0.9616
ViT-l-p16^24^^†^	0.9603	0.9533	0.9531	0.9550
T2T-VIT-12^59^‡	–	–	0.9910	–
TransResUNet^60^^†^	0.9502	0.9492	0.9484	0.9460
BPECN^61^^‡^	–	–	0.9772	–
SKAL-AlexNet^62^^‡^	–	–	0.9738	–
SKAL-ResNet18^62^^‡^	–	–	0.9952	–
SKAL-GoogleNet^62^^‡^	–	–	0.9940	–
GCSANet^63^^‡^	–	–	0.9832	–
EMTCAL^32^^‡^	–	–	0.9929	–
SF-MSFormer-ResNet18^64^^‡^	–	–	0.9935	–
LGLFormer^65^^‡^	–	–	0.9948	–
LFAGCU (ours)	**0.9966**	**0.9960**	**0.9953**	**0.9962**

Bold indicates the optimal solution, ^‡^represents the data results from the reference literature, and ^†^represents the experimental results based on the settings of this paper's parameters.

In our investigation of the few-category multi-sample dataset, specifically the RSSCN7, we selected 2240 images as training samples. Through comparative analysis (refer to Table 2), it was observed that under conditions of ample samples, the LFAGCU model exhibited outstanding performance, Pre, Rec, Acc, and F1 scores of 0.9820, 0.9820, 0.9820, and 0.9818, respectively. In contrast, the DenseNet161 model from the fully convolutional network series also demonstrated remarkable performance (Acc 0.9712, F1 0.9706), highlighting the effectiveness of deep convolutional network stacking in effectively extracting potential semantic features from images when an adequate number of samples is available. Notably, compared to the DenseNet161 model, LFAGCU exhibited a notable increase of 1.06% and 1.14% in Acc and F1 metrics, respectively, demonstrating not only the efficiency of LFAGCU in utilizing limited biases and inductive techniques but also the excellent performance of its combined GFL in considering spatial relations between fine-grained image pixels and geographical attribute features. Furthermore, compared to the recent DFAGCN and SNN-VGG-15 models, LFAGCU showed an improvement of 4.06% and 3.66% in the Acc metric, respectively. In comparison to the ViT models based on wide-range feature extraction, LFAGCU exhibited at least a 2.52% improvement across all four major metrics. These results not only demonstrate LFAGCU's strong capability in understanding the spatial relationships and intrinsic contextual cues of terrestrial entities but also confirm the effectiveness of combining local and global semantic information in two design paradigms for embedding information in collaborative learning images. When compared with the best-performing local and global paradigm model, SKAL-ResNet18^62^, LFAGCU still holds a 2.16% higher accuracy. This result is attributed to LFAGCU’s more effective feature extraction and fusion mechanisms that capture the details (local features) and overall structure (global features) in remote sensing images.

In our research involving the WHU-RS19 and UCMerced-LandUse datasets, each containing multiple categories and limited samples, approximately 42 and 80 images were selected for training in each category, respectively (refer to Tables 3 and 4). A detailed analysis from Table 3 reveals that despite the limited number of training samples, leveraging state-of-the-art fully convolutional network techniques such as Efficient-b6-1 k and Convnext-b-1 k achieved F1 scores of 99.34% and 99.01%, respectively. This highlights the significant impact of utilizing pre-trained parameters (where 1 k and 22 k represent pre-training using the ImageNet-1 K and ImageNet-22 K datasets, respectively) in enhancing the precision of deep convolutional networks, thereby achieving outstanding performance in addressing practical issues. However, it is noteworthy that overfitting issues persist in some networks, such as AlexNet, within the limited sample datasets. Figure 10 illustrates the confusion matrices of several models that achieved identical accuracy values on the WHU-RS19 dataset. Although these models demonstrate uniformity in aggregate accuracy metrics, the confusion matrices reveal nuanced variations in classification performance across different categories. On the WHU-RS19 dataset, our novel LFAGCU model demonstrated outstanding performance, achieving an Acc of 98.96% and an F1 score of 99.34%, surpassing traditional CNN networks and recent popular feature aggregation networks comprehensively. Compared to local–global paradigm models such as TransResUNet, BPECN, SF-MSFormer-ResNet18, and LGFormer, LFAGCU still displays a distinct advantage. Although the latter three models have not provided comprehensive evaluation metrics, only their accuracy, LFAGCU continues to demonstrate superior overall performance. Notably, when compared with LGFormer, which boasts an accuracy rate as high as 99.20%, LFAGCU still outperforms in terms of comprehensive capabilities. This enhanced performance can be attributed to LFAGCU’s implementation of effective structural designs and strategies, which proficiently integrate local and global features. This integration significantly boosts the model's capability to comprehend and process complex data patterns. In summary, LFAGCU has demonstrated robust potential and exceptional performance in challenging classification tasks throughout these experiments.

Furthermore, an analysis of Table 4 reveals that on the UCMerced-LandUse dataset, the LFAGCU model achieved an accuracy of 99.53% and an F1 score of 99.62%. This underscores its exceptional ability in collaborative learning and embedding information within images, particularly evident in scenarios involving small samples, unaffected by dataset sparsity or sample size limitations. Compared to the ViT network, LFAGCU shows improvements of 3.29% in accuracy and 3.46% in F1 score. Even when compared to the best-performing local–global paradigm model referenced in the literature, SKAL-ResNet18, which had an accuracy of 99.52%, LFAGCU's performance is slightly superior. The balanced performance of LFAGCU across all evaluation metrics, including precision and recall, provides a more comprehensive understanding of its capabilities, highlighting its superiority in handling complex remote sensing image tasks.

### Nonlinear transformation ablation experiment

**Figure 5:**
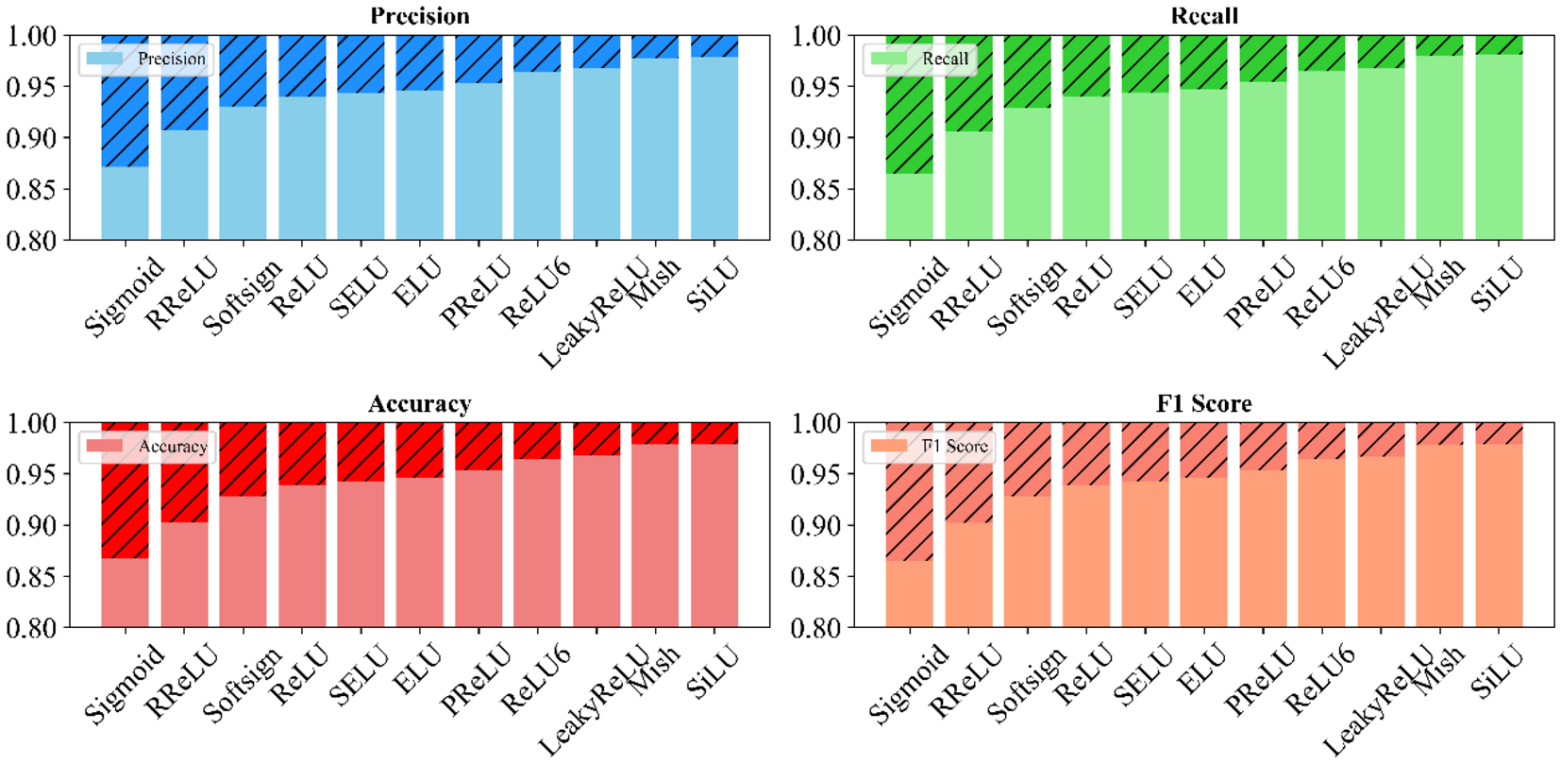
Impact of activation functions on land cover classification performance on RSSCN7.

**Figure 6:**
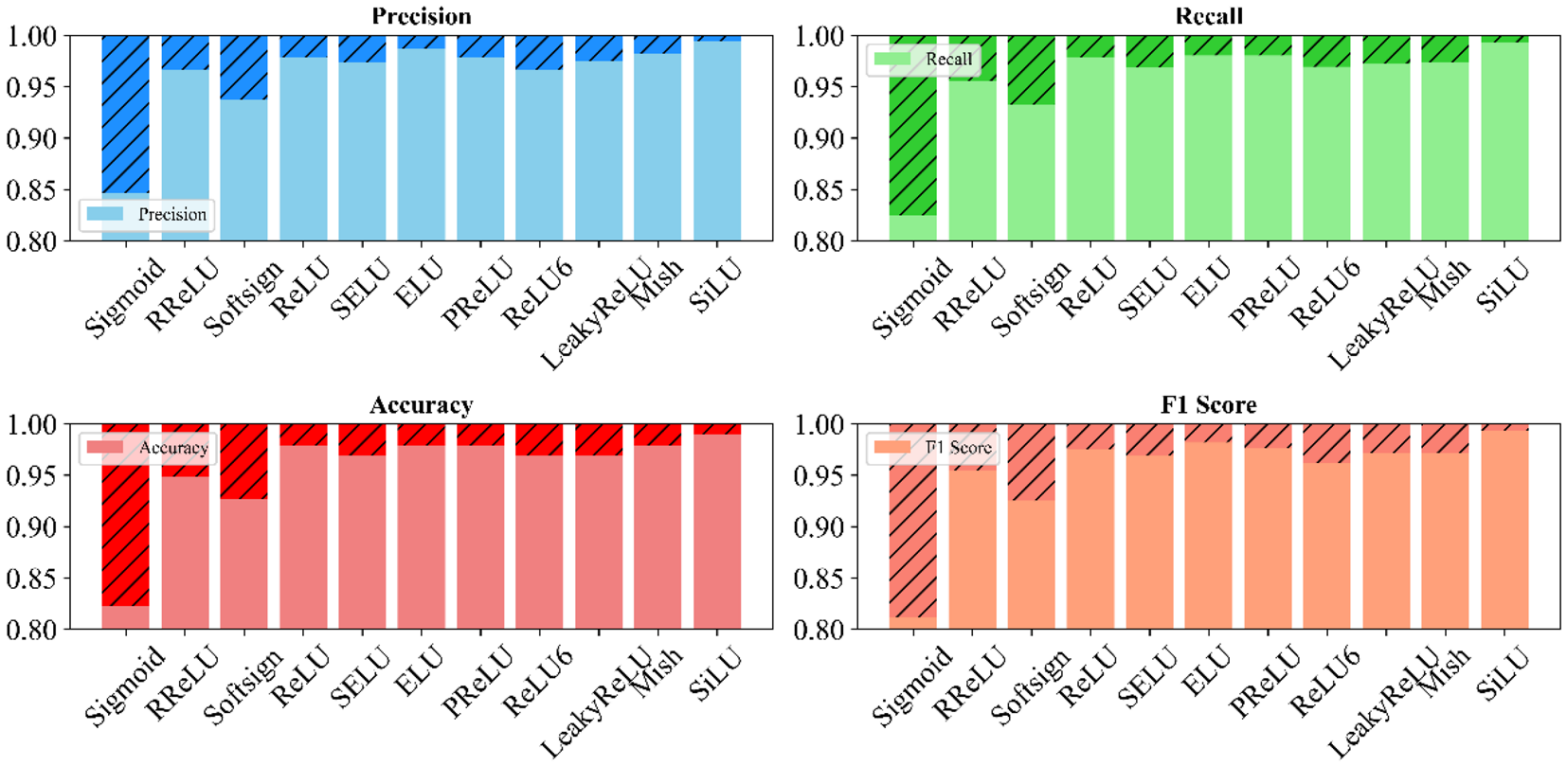
Impact of activation functions on land cover classification performance on WHU-RS19.

**Figure 7:**
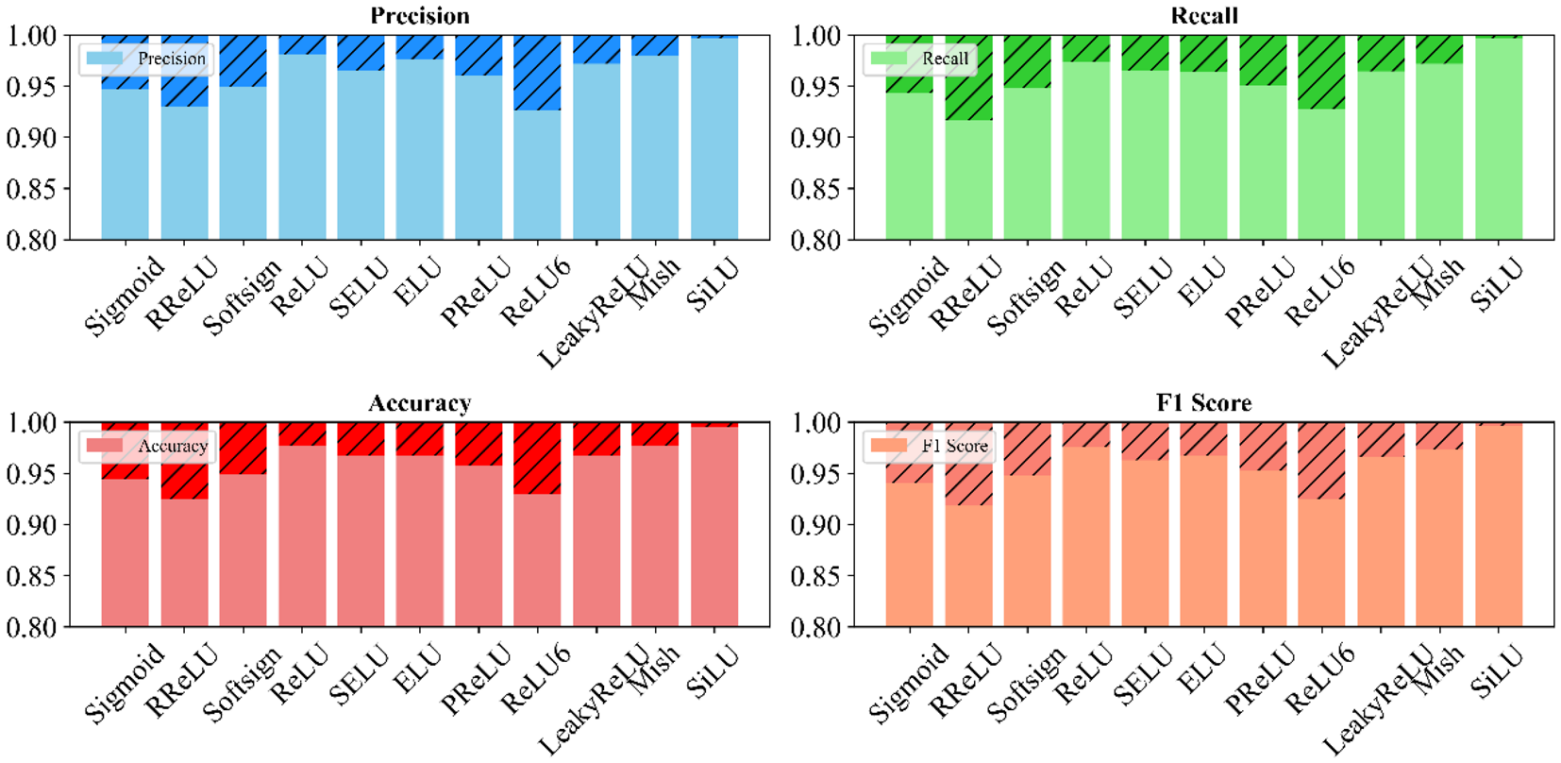
Impact of activation functions on land cover classification performance on UCMerced-LandUse.

This study analyzes various activation functions' impact on land cover classification using the LFAGCU model across three datasets: RSSCN7, WHU-RS19, and UCMerced-LandUse. Eleven activation functions: Sigmoid, RReLU, Softsign, ReLU, SELU, ELU, PReLU, ReLU6, LeakyReLU, Mish, and SiLU are evaluated (as shown in Figs. 5, 6, and 7).

Across all metrics, SiLU performed best on the RSSCN7 dataset, capturing fine-grained nuances. ELU excelled in precision, recall, and accuracy on the WHU-RS19 dataset, with SiLU demonstrating adaptability. On the UCMerced-LandUse dataset, SiLU again performed outstandingly, followed by ReLU. Overall, SiLU consistently demonstrated high-performance characteristics across datasets, providing valuable optimization insights for accurate land cover classification using the LFAGCU model.

### Comparative experiment and analysis

In this study, a particular focus on exploring the impact of different hyperparameters within the GFL module. These hyperparameters encompass:

1. Input and output channel dimensions prior to the GFL module (Into/Out GFL-channels): These parameters influence the propagation and transformation of information within the GFL module, thereby affecting feature learning and model performance.
2. Number of channels used for computing attention weights within the GFL module (Attention-channels): This parameter determines the dimensionality of each channel within the attention mechanism, thereby influencing the model's capacity to learn relationships among different channels.
3. Dimensionality of linear layers within the GFL module (GFL-dimension): This parameter determines the dimensionality of the internal linear layers of the GFL module, consequently impacting the complexity and richness of feature transformation.

The study evaluated the effects of these hyperparameters on model performance by exploring them in different model configurations (LFAGCU, LFAGCU (M), and LFAGCU (S)). These investigations were conducted through experimentation involving variations in channel dimensions and transformation sizes, as presented in the tabulated results (Table 5).

**Table 5:** LFAGCU model performance under various hyperparameter configurations.

	LFAGCU	LFAGCU (M)	LFAGCU (S)
Into/Out GFL-channels	64/160	48/96	24/80
Attention-channels	144/240	96/144	64/96
GFL-dimension	288/480	192/288	128/192
Total params	2,664,120	1,199,064	771,928
Trainable params	2,664,120	1,199,064	771,928
Total mult-adds (M)	28.27	14.48	10.54
Forward/backward pass size (MB)	3.87	3.56	3.45
Params size (MB)	10.16	4.57	2.94
Estimated total size (MB)	14.60	8.71	6.97

The upper part of the table presents data in the format of: 'Initial Layer/Final Layer'.

By observing the experimental results in Table 5, it becomes apparent that the "LFAGCU" model exhibits the highest complexity, with the greatest total parameters and parameter count. This suggests that the model possesses a more potent capability to capture intricate land cover features. However, this increase in complexity comes at the expense of computational efficiency, as indicated by its notably elevated total multiply-adds. On the other hand, while the "LFAGCU (S)" model boasts the lowest complexity, it may still retain reasonable performance within the framework of local perception and global context modeling. We note that the reduction in GFL hyperparameters corresponds to reduced model complexity and memory requirements. The "LFAGCU" model incurs the highest memory usage due to its larger parameter size, whereas the "LFAGCU (S)" model demonstrates markedly lower memory usage owing to its smaller parameter dimensions. When selecting a land cover classification model architecture, a trade-off between performance and complexity is crucial. We anticipate that the "LFAGCU" model could excel in tasks necessitating comprehensive extraction of remote sensing features. This balancing act can be tailored to specific task requirements and computational resources available.

In the data analysis presented in Table 6, there is an overall increasing trend in model performance as the hyperparameters transition from 'Min' to 'Med' and then to 'LFAGCU', aligning with the initial expectations. This trend indicates that, in the context of VHR remote sensing image land cover classification tasks, employing larger model configurations generally leads to improved classification performance. Whether considering few-category multi-sample datasets (RSSCN7) or multi-category few-sample datasets (WHU-RS19 and UCMerced-LandUse), LFAGCU consistently exhibits the highest precision, recall, accuracy, and F1 score. This observation suggests that adopting larger model dimensions in terms of channels and GFL components contributes to a more reliable capture of intricate land cover patterns, thereby enhancing the performance of VHR remote sensing image classification.

**Table 6:** Performance of LFAGCU with different hyperparameters on different datasets.

		Pre	Rec	Acc	F1
RSSCN7	LFAGCU (S)	0.9510	0.9504	0.9496	0.9486
	LFAGCU (M)	0.9615	0.9612	0.9604	0.9611
	LFAGCU	0.9820	0.9820	0.9820	0.9818
WHU-RS19	LFAGCU (S)	0.9658	0.9675	0.9688	0.9645
	LFAGCU (M)	0.9854	0.9829	0.9792	0.9828
	LFAGCU	0.9942	0.9934	0.9896	0.9934
UCMerced-LandUse	LFAGCU (S)	0.9588	0.9604	0.9624	0.9587
	LFAGCU (M)	0.9807	0.9775	0.9812	0.9767
	LFAGCU	0.9942	0.9934	0.9896	0.9934

**Figure 8:**
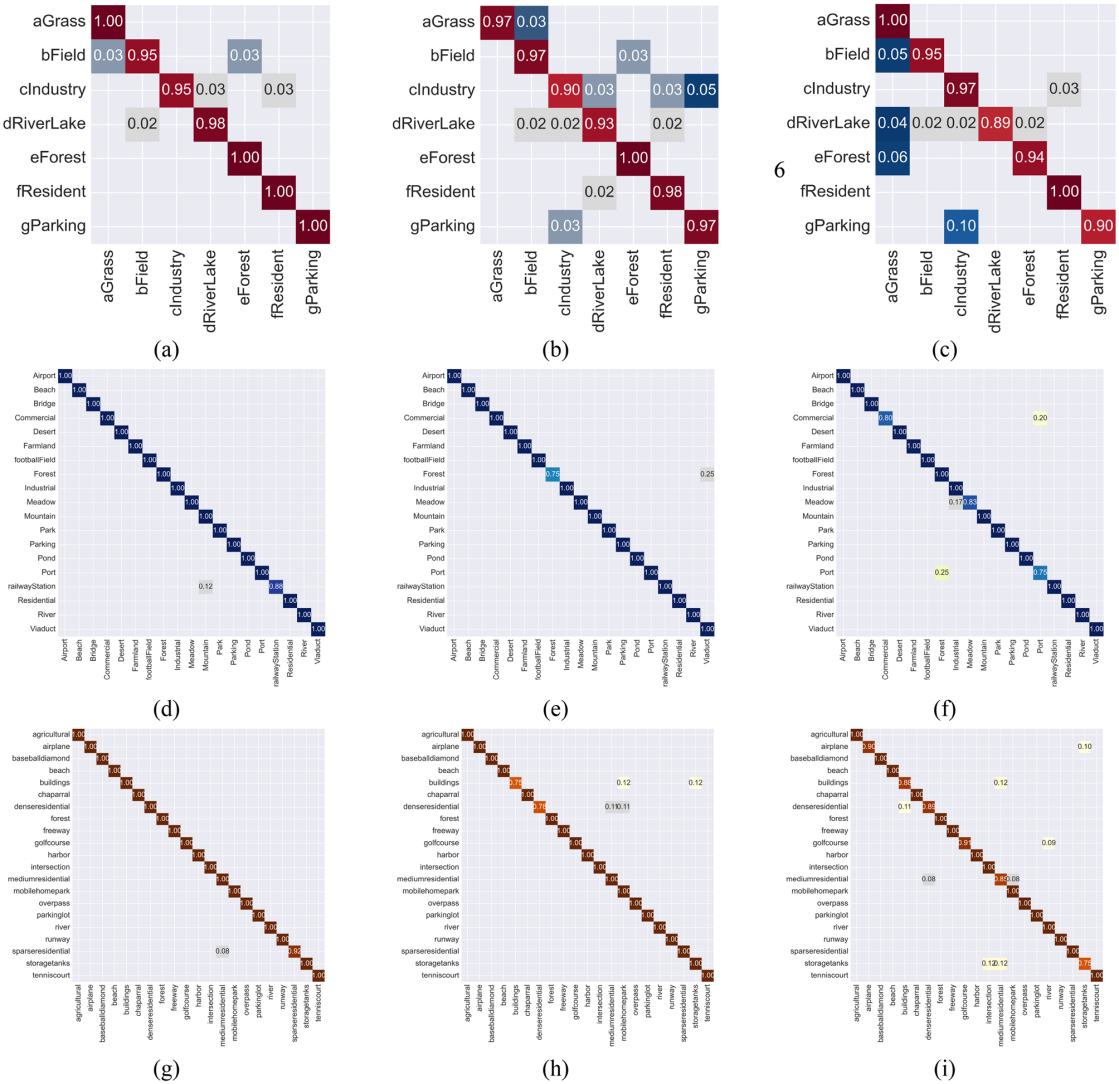
Visualization of confusion matrices for the three datasets. RSSCN7 represented as (**a**), (**b**), and (**c**); WHU-RS19 denoted as (**d**), (**e**), and (**f**); and UCMerced-LandUse depicted in (**g**), (**h**), and (**i**). Within these visualizations, (**a**), (**d**), and (**g**) correspond to the application of the LFAGCU method; (**b**), (**e**), and (**h**) relate to the utilization of LFAGCU (M) method; while (**c**), (**f**), and (**i**) illustrate the application of LFAGCU (S) method.

**Figure 9:**
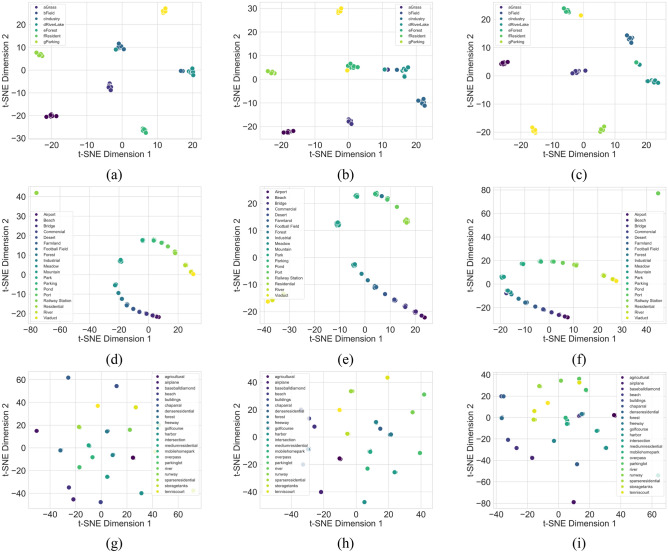
Visualization of t-SNE for the three datasets. RSSCN7 represented as (**a**), (**b**), and (**c**); WHU-RS19 denoted as (**d**), (**e**), and (**f**); and UCMerced-LandUse depicted in (**g**), (**h**), and (**i**). Within these visualizations, (**a**), (**d**), and (**g**) correspond to the application of the LFAGCU method; (**b**), (**e**), and (**h**) relate to the utilization of LFAGCU (M) method; while (**c**), (**f**), and (**i**) illustrate the application of LFAGCU (S) method.

**Figure 10:**
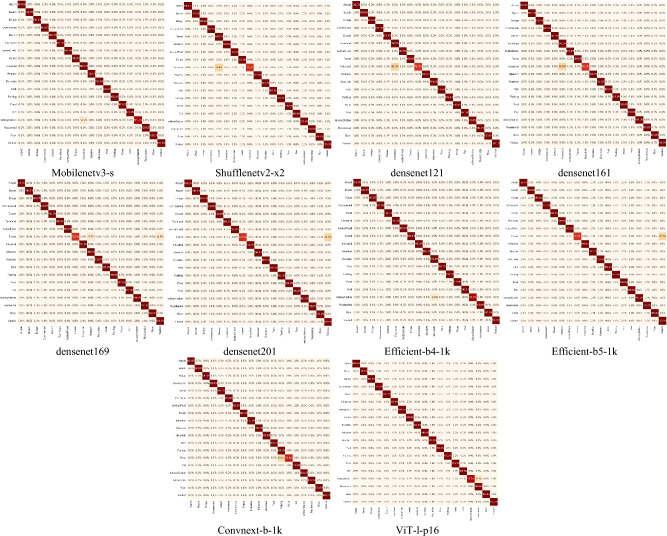
Confusion matrices comparing multiple models on the WHU-RS19 dataset.

This superior performance is further validated through two visualization techniques: confusion matrix and t-SNE analysis. From observations in Fig. 8, it is evident that LFAGCU exhibits the most substantial and numerous true positive values. In Fig. 9, the scatter plot resulting from t-SNE dimensionality reduction displays each sample's position in the visualization space, reflecting its position in the reduced feature space. Notably, LFAGCU's scatter plot exhibits the most distinct clustering within each class, affirming that the adoption of larger model dimensions in terms of channels and GFL components effectively captures intricate land cover patterns, significantly enhancing classification performance for high-resolution remote sensing images (Fig. 10).

## Conclusion

To address the challenge of high-resolution remote sensing image land cover classification, this paper proposes an innovative dual-feature learning paradigm model named LFAGCU. Firstly, we designed a multi-channel and multi-dimensional local feature extractor, focusing on deep exploration of local details and semantic features across various spatial dimensions in remote sensing images. This step aims to comprehensively capture subtle land object characteristics in images, enhancing classification accuracy. Secondly, we introduced the GFL module aimed at abstracting global features and contextual semantic information of remote sensing images. This module aids in understanding the overall background and context of the images, thereby enhancing the model's ability to recognize the entire image. Finally, we conducted extensive comparative and ablation experiments on three open-source datasets, including RDDCN7, WHU-RS19, and UCMerced-LandUse. The results demonstrate LFAGCU's significant competitiveness in land cover classification, maintaining a leading position and exhibiting robust generalization capabilities. In future work, we intend to thoroughly explore the influence of local and global information captured at various receptive field sizes on the attribute information of land objects. Our goal is to refine and enhance current models, bolstering their capacity to efficiently process and analyze remote sensing imagery. This investigation is pivotal in improving model robustness and adaptability, ensuring that they perform consistently across a broad spectrum of domains and applications within remote sensing tasks. By optimizing the interplay between local detail and global context, we aspire to push the boundaries of what these sophisticated models can achieve in diverse settings, from urban landscapes to natural environments.

## Data Availability

The RSSCN7 dataset^38^ analysed during the current study is accessible at the link: https://github.com/palewithout/RSSCN7. The WHU-RS19 dataset^39^ analysed during the current study is accessible at the link: https://captain-whu.github.io/BED4RS/#. The UCMerced-LandUse dataset^40^ analysed during the current study is accessible at the link: http://weegee.vision.ucmerced.edu/datasets/landuse.html.
